# Non-visualization of the Gallbladder on Ultrasound: Magnetic Resonance Cholangiopancreatography (MRCP) Confirmation of Congenital Gallbladder Agenesis

**DOI:** 10.7759/cureus.107005

**Published:** 2026-04-14

**Authors:** Owais H Ghulman, Maan B Ghulman, Reem Mimish, Mohammed A Krimly

**Affiliations:** 1 College of Medicine and Surgery, Faculty of Medicine, King Abdulaziz University, Jeddah, SAU; 2 Radiology, King Abdulaziz University Hospital, Jeddah, SAU; 3 Abdominal Imaging, Faculty of Medicine, King Abdulaziz University, Jeddah, SAU; 4 Radiology, Faculty of Medicine, King Abdulaziz University, Jeddah, SAU

**Keywords:** congenital biliary anomaly, gallbladder agenesis, magnetic resonance cholangio-pancreatography (mrcp), non-visualized gallbladder, right upper abdominal pain

## Abstract

Gallbladder agenesis is a rare congenital biliary anomaly that may mimic gallbladder pathology both clinically and radiologically. On ultrasound, it may be misinterpreted as a contracted gallbladder or cholelithiasis, potentially leading to diagnostic confusion and unnecessary surgical exploration.

We report the case of a 23-year-old female who presented with right upper quadrant abdominal pain and whose initial ultrasound suggested gallstones. Because the gallbladder was not clearly visualized, magnetic resonance cholangiopancreatography (MRCP) was performed for further evaluation. MRCP demonstrated the absence of the gallbladder with otherwise normal biliary anatomy, confirming congenital gallbladder agenesis. The echogenic foci initially interpreted as gallstones were later presumed to represent artifactual echoes or adjacent soft tissue/bowel interfaces rather than true cholelithiasis. The unique contribution of this case lies in highlighting a common diagnostic pitfall in which non-visualization of the gallbladder with false-positive sonographic suspicion of stones may prompt unnecessary operative planning if biliary anatomy is not clarified. This case highlights the diagnostic importance of MRCP when the gallbladder is not visualized on ultrasound and emphasizes its role in avoiding unnecessary surgical intervention in selected equivocal cases.

## Introduction

Gallbladder agenesis is a rare congenital anomaly with an estimated incidence of 10-65 per 100,000 individuals [1]. It results from the failure of development of the cystic bud from the hepatic diverticulum during early embryogenesis [1,2]. Although some individuals remain asymptomatic, others present with symptoms that mimic biliary colic or gallstone disease, often creating diagnostic uncertainty [2,3].

Ultrasound is typically the first-line imaging modality in the evaluation of right upper quadrant pain; however, non-visualization of the gallbladder may be misinterpreted as a contracted gallbladder, chronic cholecystitis, or obscured anatomy [2,4]. This may result in an incorrect presumptive diagnosis and, in some cases, unnecessary surgical exploration [2,3]. Magnetic resonance cholangiopancreatography (MRCP) plays an important role in confirming the diagnosis by accurately delineating the biliary tree and excluding an ectopic gallbladder or post-surgical absence [4,5].

Although the role of MRCP in confirming gallbladder agenesis is already well established, the novelty of this case lies in demonstrating a practical diagnostic pitfall: false sonographic suspicion of cholelithiasis in the setting of a non-visualized gallbladder. This case therefore emphasizes not merely the diagnosis itself but the importance of clarifying biliary anatomy before operative decision-making when ultrasound findings are equivocal [6,7].

This case report aims to highlight the role of MRCP in confirming congenital gallbladder agenesis when ultrasound findings are inconclusive and to emphasize its value in preventing unnecessary surgical intervention.

## Case presentation

A 23-year-old female presented with intermittent right upper quadrant abdominal pain consistent with biliary-type colic. She reported that the pain had been occurring for several months, although she was unable to recall the exact duration. The pain was described as recurrent and was noted to worsen after fatty meals. It was associated with nausea but not with vomiting, fever, or jaundice. There was no history of prior abdominal surgery and no known personal history of hepatobiliary disease. Family history was notable for gallbladder disease in her paternal grandmother. She denied oral contraceptive use. Pregnancy test at the time of presentation was negative. Vitals were within normal limits.

On presentation, the patient was hemodynamically stable and afebrile. Physical examination revealed mild right upper quadrant tenderness without guarding or rebound. No palpable abdominal mass or jaundice was noted.

Initial differential considerations included biliary colic secondary to cholelithiasis, chronic cholecystitis with contracted gallbladder, biliary dyskinesia, choledocholithiasis, and less likely non-biliary causes of right upper quadrant pain [8,9].

**Figure 1:**
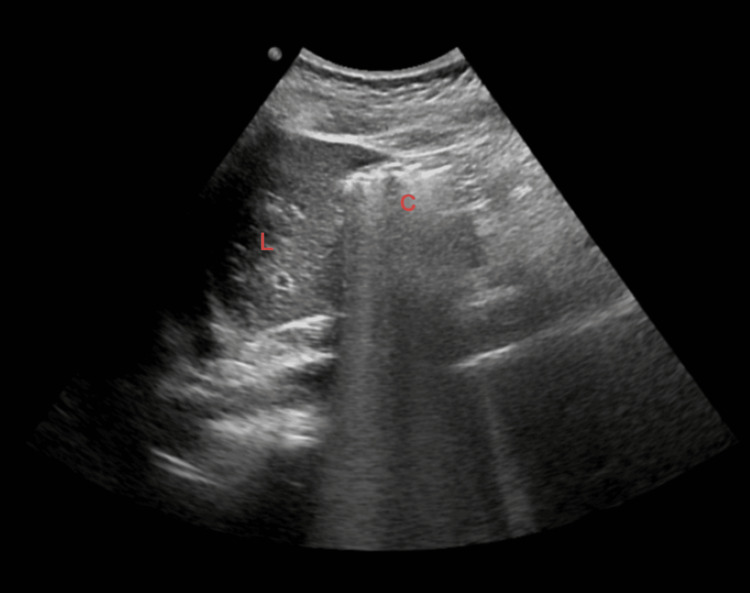
Initial ultrasound Abdominal ultrasound demonstrating non-visualization of the gallbladder in the gallbladder fossa. The echogenic focus, initially suspected to represent gallstones, was later determined not to correspond to a true gallbladder structure (L = liver, C = colon).

Initial transabdominal ultrasound demonstrated echogenic foci suspicious for gallstones; however, the gallbladder was not clearly visualized (Figure 1), raising the possibility of a contracted or obscured gallbladder [2,4].

Laboratory investigations were within normal limits, including total protein 74 g/L, albumin 48 g/L, aspartate aminotransferase (AST) 18 U/L, alkaline phosphatase (ALP) 62 U/L, gamma-glutamyl transferase (GGT) 11 U/L, and total bilirubin 10 μmol/L. Alanine aminotransferase (ALT) was also within normal limits. Complete blood count (CBC) and C-reactive protein (CRP) were unremarkable. Overall, there was no clinical or biochemical evidence of acute cholecystitis, biliary obstruction, pancreatitis, or hepatocellular injury.

To further evaluate the non-visualized gallbladder, magnetic resonance imaging (MRI) of the abdomen was performed using a 1.5 Tesla system. Sequences included axial T1-weighted in-phase and out-of-phase imaging, multiplanar T2-weighted imaging, diffusion-weighted imaging, and fat-suppressed T1-weighted images obtained before and after intravenous gadolinium administration. MRCP sequences were also acquired [7].

**Figure 2:**
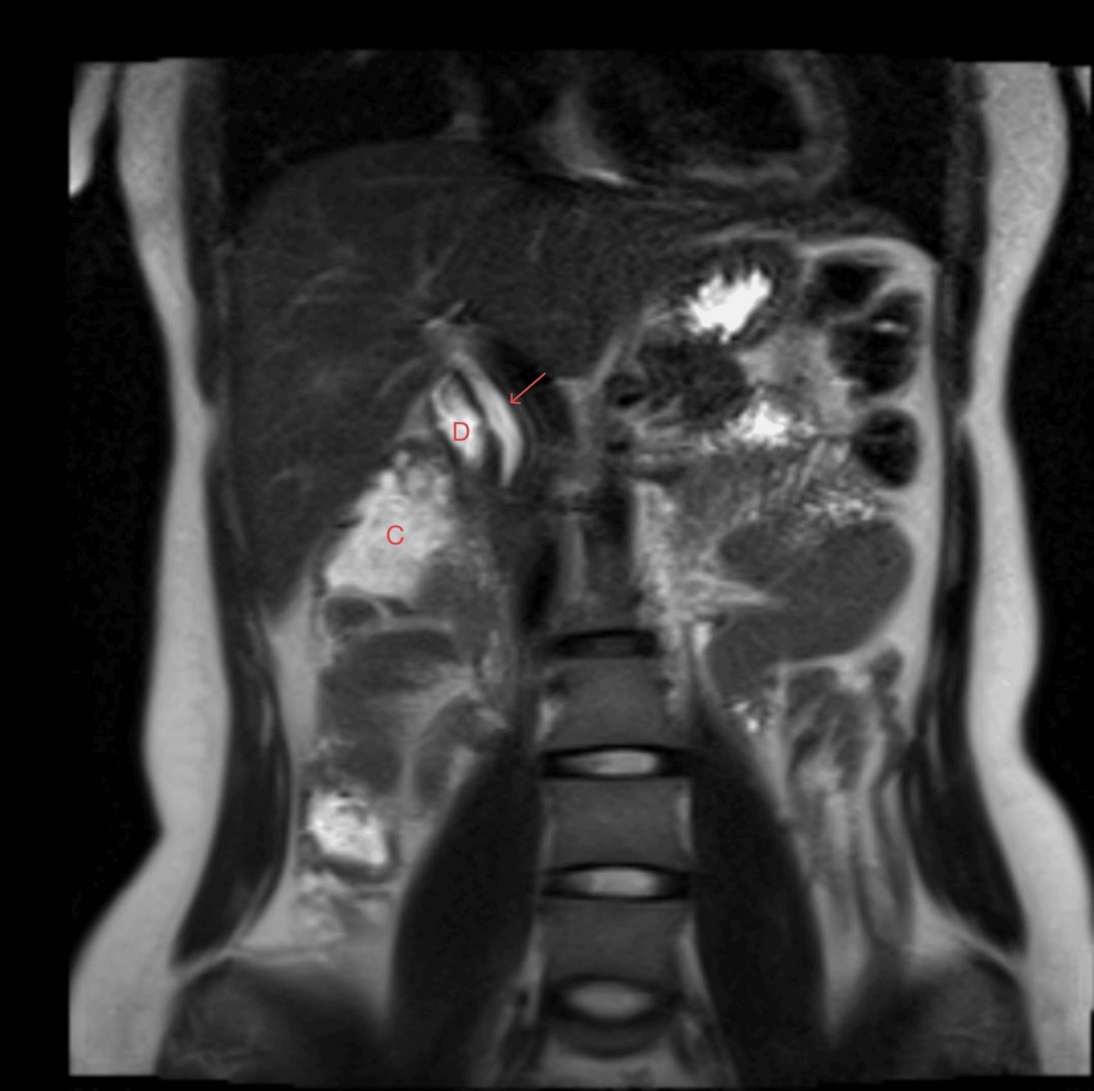
Coronal T2-weighted MRI Coronal T2-weighted MRI of the abdomen showing non-visualization of the gallbladder in the expected anatomical location (arrow, CBD), with adjacent duodenum (D) and colon (C). MRI: magnetic resonance imaging; CBD: common bile duct

**Figure 3:**
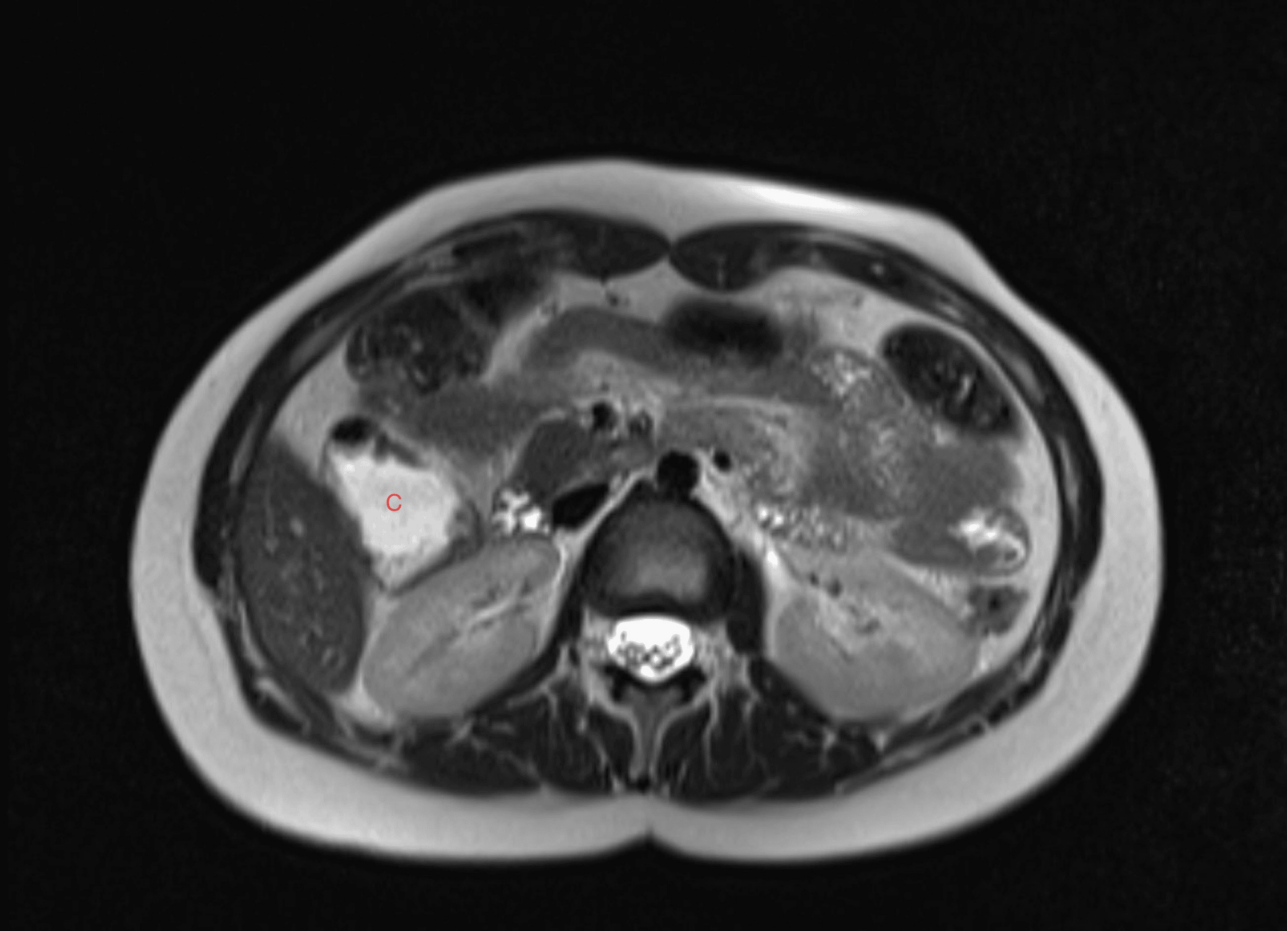
Axial T2-weighted MRI Axial T2-weighted MRI demonstrating absence of the gallbladder, with bowel loops occupying the expected gallbladder fossa (C = colon). MRI: magnetic resonance imaging

Coronal and axial T2-weighted MRI demonstrated the absence of the gallbladder in its expected anatomical location (Figures 2-3).

**Figure 4:**
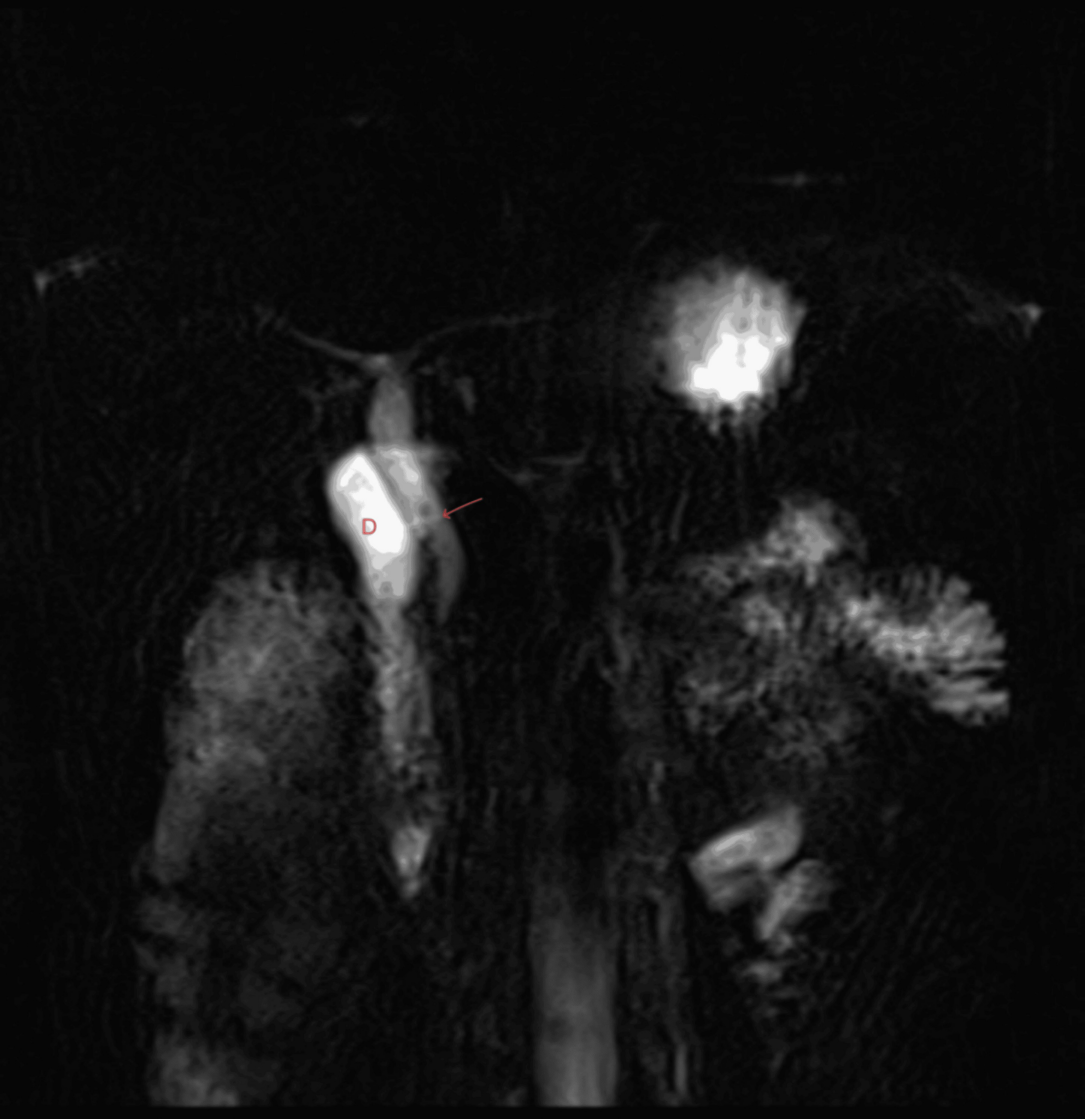
Coronal MRCP Coronal MRCP confirming absence of the gallbladder and cystic duct, showing normal intrahepatic ducts and a mildly prominent common bile duct. MRCP: magnetic resonance cholangiopancreatography

MRCP confirmed these findings, demonstrating normal intrahepatic bile ducts and a mildly prominent common bile duct measuring 6 mm. The gallbladder was not visualized in the expected anatomical location [5]. No ectopic gallbladder was identified, and there was no evidence of biliary obstruction or choledocholithiasis (Figure 4).

In retrospect, the echogenic foci seen on ultrasound were presumed to reflect artifactual echoes, reverberation artifact, adjacent bowel gas, or echogenic soft tissue interfaces within the gallbladder fossa, rather than true gallstones [10]. The remainder of the examination was unremarkable. The imaging findings were consistent with congenital gallbladder agenesis. The patient was managed conservatively with symptomatic treatment, with subsequent resolution of abdominal pain and no need for surgical intervention [11].

## Discussion

Gallbladder agenesis is a rare developmental anomaly caused by failure of the cystic bud to form during the fourth week of embryologic development [1]. It may occur as an isolated anomaly or, less commonly, in association with other congenital malformations [1]. Clinical presentation is variable, ranging from incidental asymptomatic detection to symptoms resembling gallstone disease [2,3].

The clinical value of the present case lies not solely in the rarity of gallbladder agenesis but in the misleading sonographic impression it created. In routine practice, a symptomatic patient with right upper quadrant pain and ultrasound findings suggestive of gallstones is far more likely to be presumed to have common gallbladder pathology than a congenital anomaly. This case, therefore, highlights an important real-world diagnostic trap in which non-visualization of the gallbladder with apparent echogenic foci may falsely suggest cholelithiasis and potentially lead to inappropriate operative planning if the biliary anatomy is not clarified [6,7].

The exact mechanism of biliary-type pain in symptomatic patients with gallbladder agenesis remains uncertain. Proposed explanations include biliary dyskinesia, sphincter of Oddi dysfunction, altered biliary dynamics, or compensatory changes in the extrahepatic biliary tree, all of which may produce symptoms resembling biliary colic despite congenital absence of the gallbladder [4].

Ultrasound remains the primary imaging modality for evaluating right upper quadrant pain, but it has recognized limitations in this setting. Non-visualization of the gallbladder may result from bowel gas, a contracted gallbladder, overlying structures, or technical factors [2,4]. In such cases, the absent gallbladder may be incorrectly interpreted as a shrunken fibrotic gallbladder, chronic cholecystitis, or gallstones within a contracted lumen [2,4,10]. In the present case, the initial sonographic impression suggested cholelithiasis because of echogenic foci identified in the gallbladder fossa; however, subsequent MRCP demonstrated congenital absence of the gallbladder. In retrospect, these echogenic foci were most likely related to reverberation artifact, adjacent bowel gas, or echogenic interfaces from periportal or surrounding soft tissues, rather than true gallstones. This underscores the importance of interpreting echogenic foci cautiously when the gallbladder itself is not confidently visualized.

When the gallbladder is not visualized on ultrasound, the differential diagnosis is broader than a contracted gallbladder alone. It includes chronic cholecystitis with a fibrotic or shrunken gallbladder, a postprandial collapsed gallbladder, ectopic or intrahepatic gallbladder, hypoplastic gallbladder, porcelain gallbladder, post-cholecystectomy state, and technical non-visualization related to bowel gas or body habitus, in addition to the rare possibility of congenital gallbladder agenesis [2,4,10]. Recognition of these possibilities is important because the sonographic appearance may otherwise be overinterpreted in favor of gallstone disease.

Historically, many cases of gallbladder agenesis were diagnosed only during surgical exploration, often following an incorrect preoperative diagnosis of gallbladder disease [1-3]. Several reports have emphasized that failure to recognize this entity preoperatively may expose patients to unnecessary operative intervention [2,3,7,8].

Cross-sectional imaging plays a decisive role when ultrasound findings are equivocal. MRCP is regarded as the most reliable non-invasive modality for confirming gallbladder agenesis because it provides detailed delineation of the biliary tree and allows exclusion of ectopic gallbladder, biliary obstruction, and other structural abnormalities [4,5]. The significance of this case is therefore not to establish MRCP as a novel diagnostic tool, but to reinforce its practical value in resolving equivocal sonographic findings before operative management is considered [6,7].

The mildly prominent common bile duct (6 mm) is also noteworthy in this case, particularly in a young patient without prior cholecystectomy. Mild compensatory dilation of the common bile duct has been reported in gallbladder agenesis and may reflect altered bile storage dynamics or chronic functional adaptation of the extrahepatic biliary system in the absence of a gallbladder reservoir [6,9]. In the present case, this finding was not associated with intrahepatic ductal dilatation, choledocholithiasis, or biochemical evidence of obstruction, supporting a non-obstructive explanation.

Importantly, MRCP should not be interpreted as a mandatory next step for every patient with biliary-type pain and a non-visualized gallbladder on ultrasound, particularly in resource-limited settings where gallstone disease remains far more prevalent than gallbladder agenesis. Rather, MRCP should be considered selectively when the ultrasound findings are discordant or equivocal, when the gallbladder cannot be confidently identified, when laboratory findings do not support acute inflammatory or obstructive biliary disease, or when surgical intervention is being considered despite uncertain anatomy [5,11]. A practical approach in such cases is to first confirm adequate fasting status and reassess ultrasound findings; if the gallbladder remains non-visualized or anatomy is still uncertain, cross-sectional biliary imaging such as MRCP should be considered before surgery. In such contexts, MRCP may help avoid unnecessary laparoscopy and improve preoperative diagnostic confidence.

Other imaging and functional studies may be less reliable in this context. Prior reports have noted that hepatobiliary scintigraphy may be misleading, as non-visualization of the gallbladder can mimic cystic duct obstruction rather than congenital absence [6,7,11]. For this reason, failure to identify the gallbladder on ultrasound should not automatically be interpreted as inflammatory or obstructive gallbladder disease in the absence of corroborating findings.

In the present case, MRCP was critical in establishing the diagnosis after inconclusive ultrasound findings. It confirmed non-visualization of the gallbladder while demonstrating otherwise normal biliary anatomy, thereby excluding obstructive pathology and preventing unnecessary surgical exploration [5].

Overall, this case reinforces the need for careful correlation between ultrasound findings, clinical presentation, laboratory data, and cross-sectional imaging before operative intervention is pursued. Its main contribution is to illustrate how a seemingly common ultrasound impression of gallstones may in fact conceal a rare anatomic diagnosis when the gallbladder is not confidently visualized [6,7,10].

## Conclusions

Gallbladder agenesis should be considered in the differential diagnosis when the gallbladder is not confidently visualized on ultrasound, particularly when the clinical, biochemical, and sonographic findings are discordant. Although MRCP is already an established diagnostic modality for confirming gallbladder agenesis, this case highlights its practical value in clarifying equivocal ultrasound findings and preventing unnecessary surgical intervention. A pragmatic diagnostic approach in such cases is to confirm appropriate fasting status, reassess ultrasound interpretation, and consider MRCP before operative management when biliary anatomy remains uncertain. In our patient, accurate imaging-based diagnosis allowed conservative treatment, with complete symptom resolution and avoidance of unnecessary surgery.
